# Supplementary material for: Phylogeny of Rhynchium and Its Related Genera (Hymenoptera: Eumeninae) Based on Universal Single-Copy Orthologs and Ultraconserved Elements
Source: Insects. 2023 Sep 20;14(9):775. doi: 10.3390/insects14090775 (PMC10532281; doi:10.3390/insects14090775)
Supplement: Supplementary file 1 [file insects-14-00775-s001.zip › Supplementary_S2_tree_topologies.pdf]

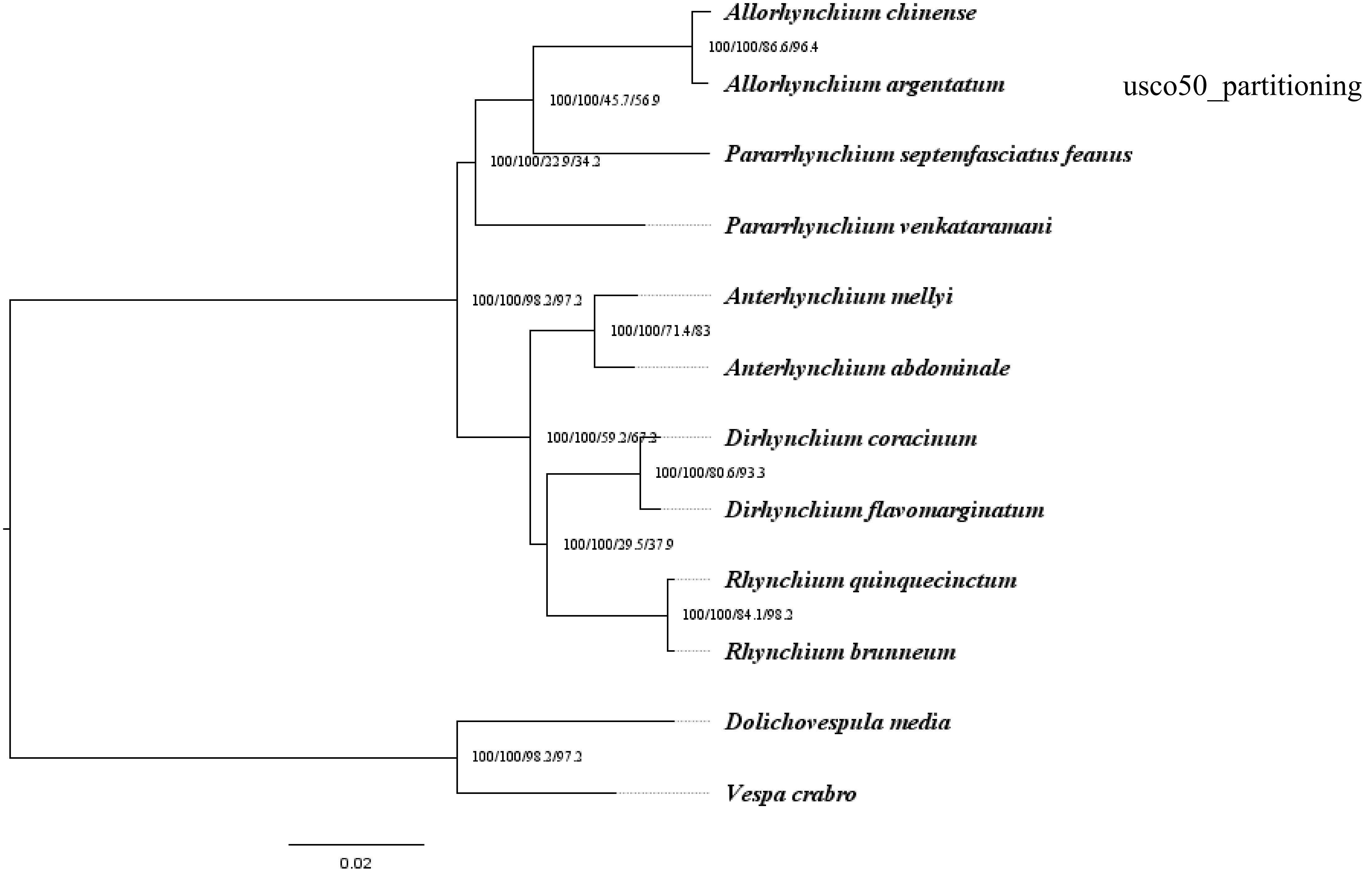
Allorhynchium chinense
100/100/86.6/96.4
Allorhynchium argentatum
usco50_partitioning
100/100/45.7/56.9
Pararrhynchium septemfasciatus feanus
100/100/22.9/34.2
Pararrhynchium venkataramani
100/100/98.2/97.2
Anterhynchium mellyi
100/100/71.4/83
Anterhynchium abdominale
100/100/59.2/67.2
Dirhynchium coracinum
100/100/80.6/93.3
Dirhynchium flavomarginatum
100/100/29.5/37.9
Rhynchium quinquecinctum
100/100/84.1/98.2
Rhynchium brunneum
Dolichovespula media
100/100/98.2/97.2
Vespa crabro
0.02

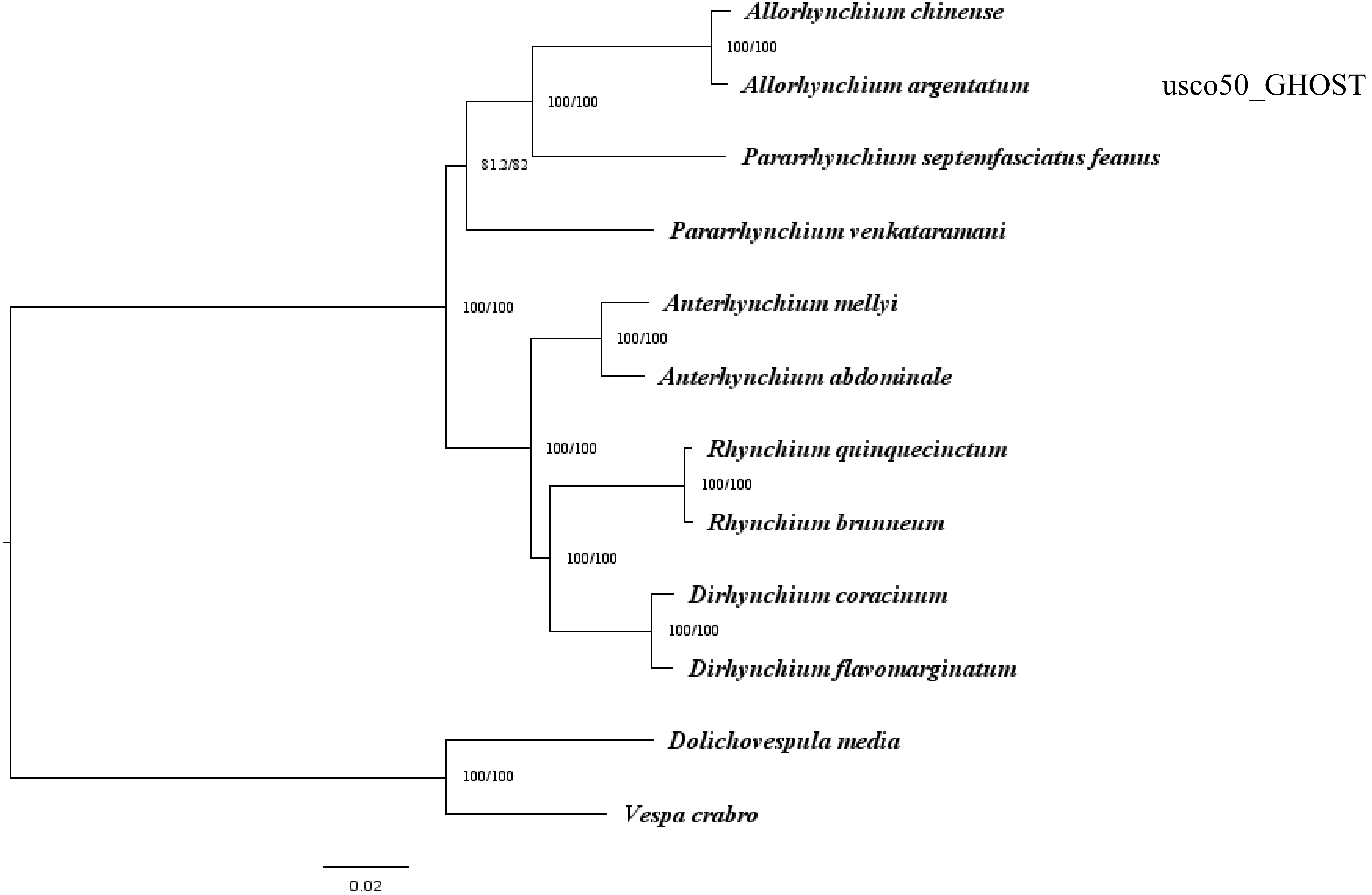
usco50_GHOST
Allorhynchium chinense
Allorhynchium argentatum
Pararrhynchium septemfasciatus feanus
Pararrhynchium venkataramani
Anterhynchium mellyi
Anterhynchium abdominale
Rhynchium quinquecinctum
Rhynchium brunneum
Dirhynchium coracinum
Dirhynchium flavomarginatum
Dolichovespula media
Vespa crabro
100/100
100/100
81.2/82
100/100
100/100
100/100
100/100
100/100
100/100
100/100
0.02

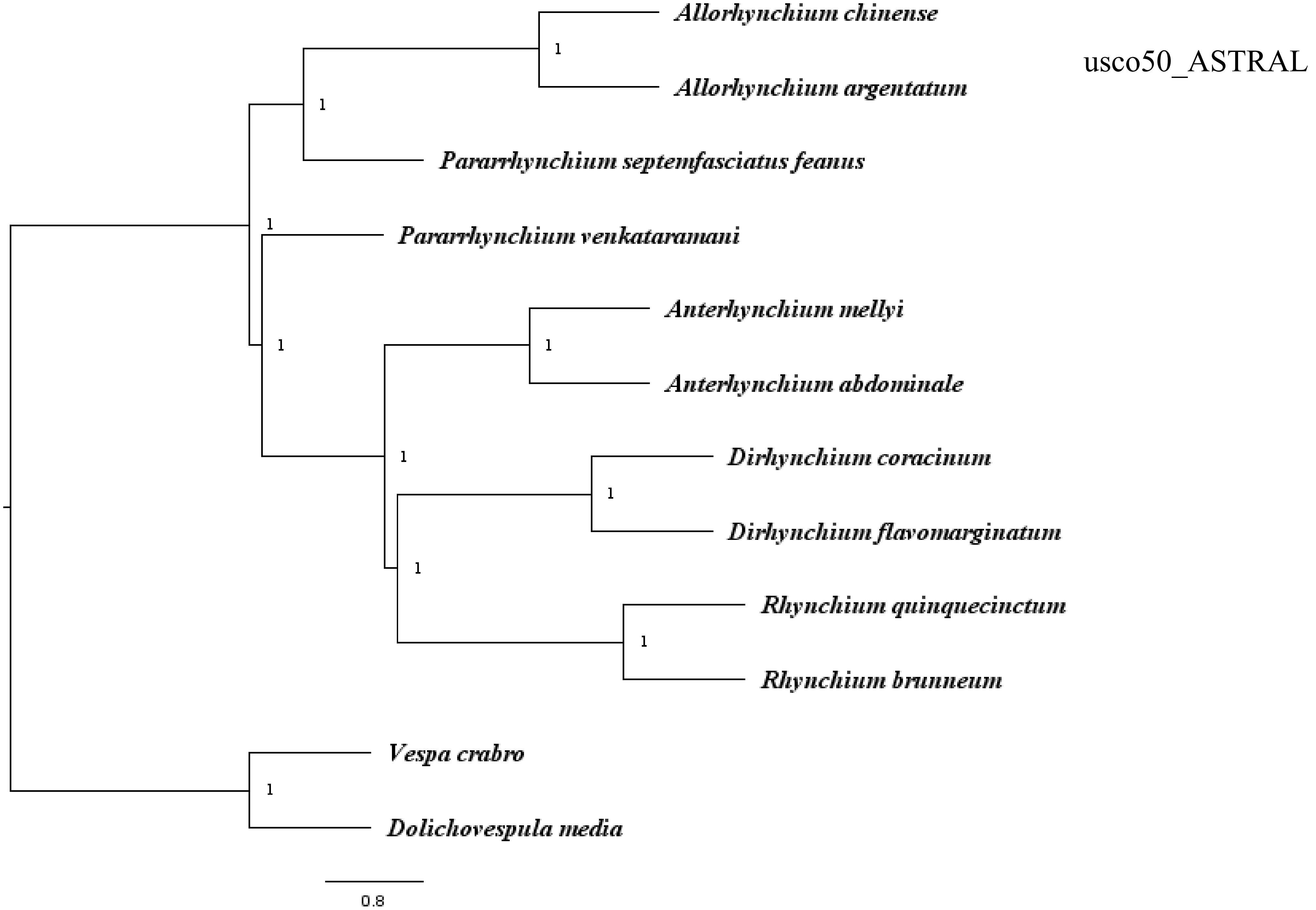
usco50_ASTRAL
Allorhynchium chinense
Allorhynchium argentatum
Pararrhynchium septemfasciatus feanus
Pararrhynchium venkataramani
Anterhynchium mellyi
Anterhynchium abdominale
Dirhynchium coracinum
Dirhynchium flavomarginatum
Rhynchium quinquecinctum
Rhynchium brunneum
Vespa crabro
Dolichovespula media
1
0.8

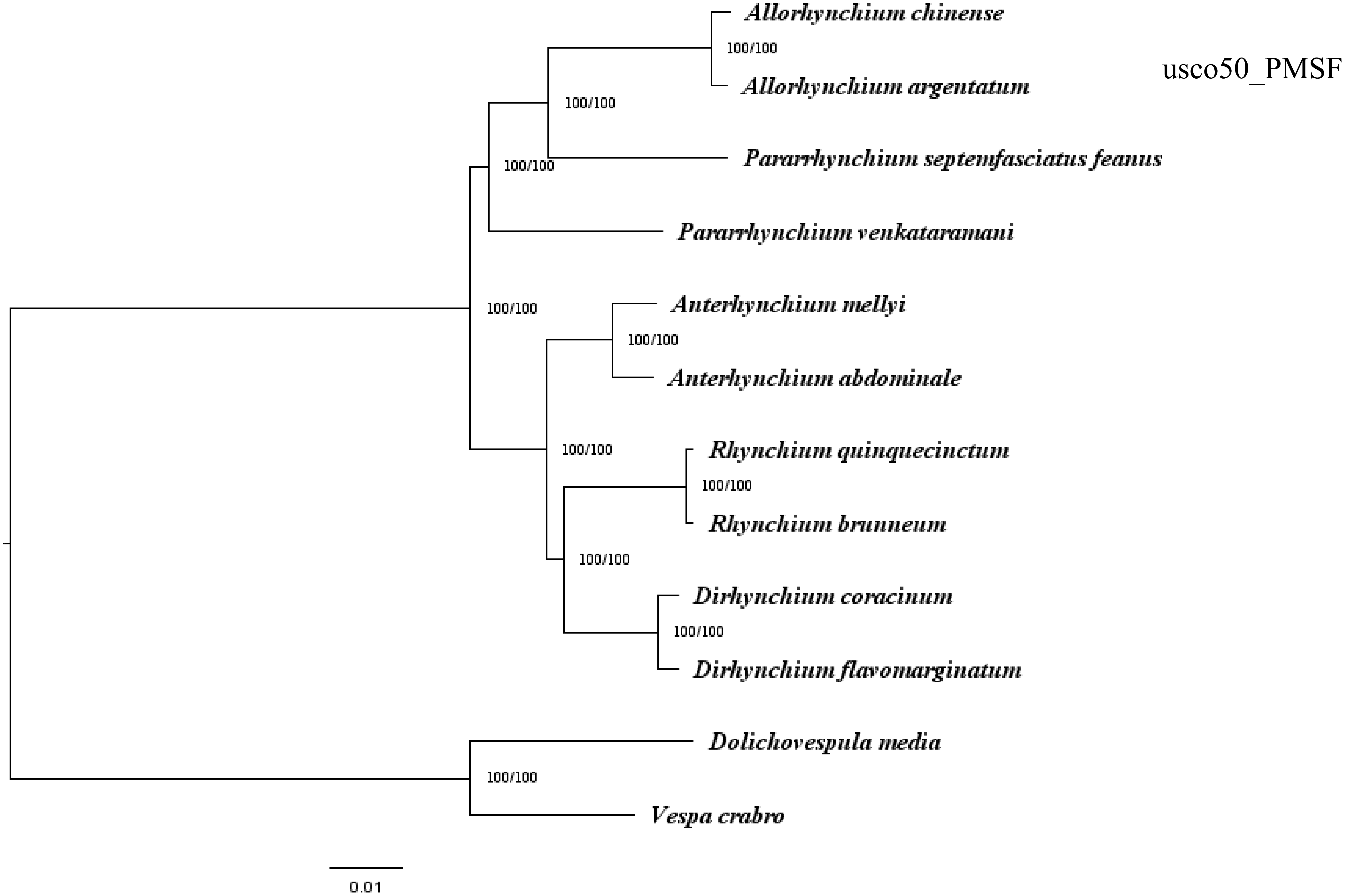

usco50_PMSF
Allorhynchium chinense
100/100
Allorhynchium argentatum
100/100
Pararrhynchium septemfasciatus feanus
100/100
Pararrhynchium venkataramani
100/100
Anterhynchium mellyi
100/100
Anterhynchium abdominale
100/100
Rhynchium quinquecinctum
100/100
Rhynchium brunneum
100/100
Dirhynchium coracinum
100/100
Dirhynchium flavomarginatum
Dolichovespula media
100/100
Vespa crabro
0.01

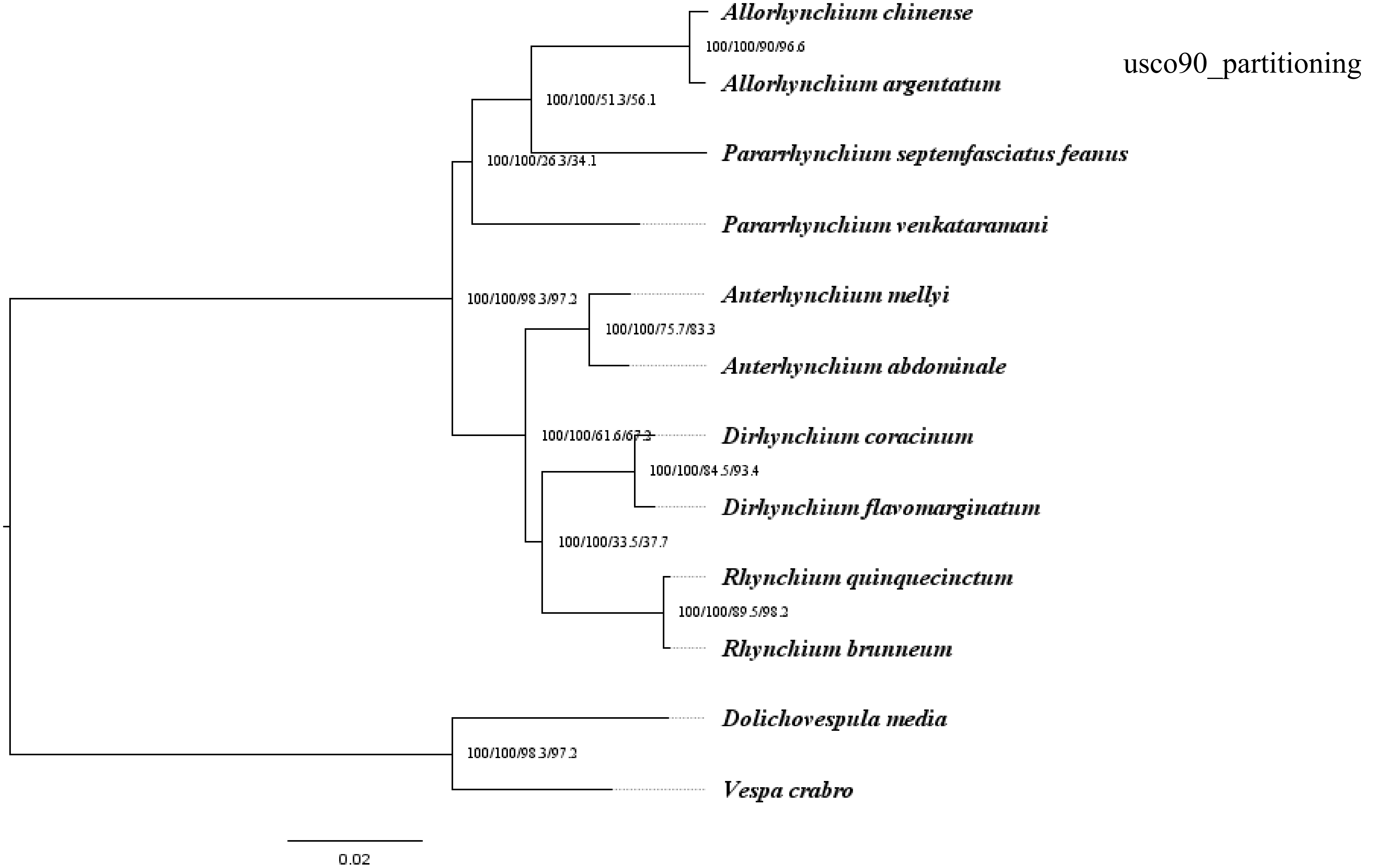
usco90_partitioning
Allorhynchium chinense
100/100/90/96.6
Allorhynchium argentatum
100/100/51.3/56.1
Pararrhynchium septemfasciatus feanus
100/100/26.3/34.1
Pararrhynchium venkataramani
100/100/98.3/97.2
Anterhynchium mellyi
100/100/75.7/83.3
Anterhynchium abdominale
100/100/61.6/67.3
Dirhynchium coracinum
100/100/84.5/93.4
Dirhynchium flavomarginatum
100/100/33.5/37.7
Rhynchium quinquecinctum
100/100/89.5/98.2
Rhynchium brunneum
Dolichovespula media
100/100/98.3/97.2
Vespa crabro
0.02

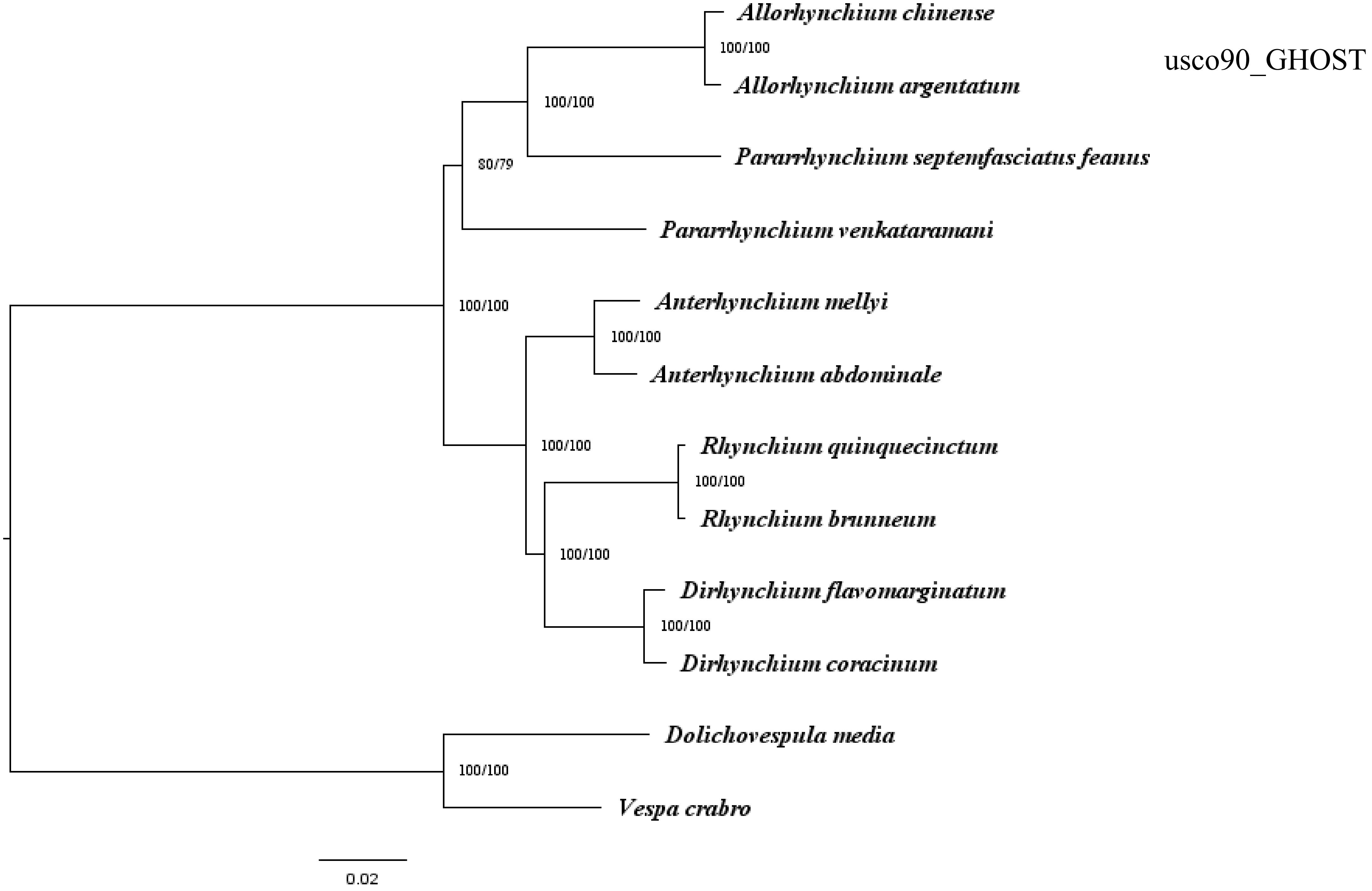
Allorhynchium chinense
100/100
Allorhynchium argentatum
100/100
Pararrhynchium septemfasciatus feanus
80/79
Pararrhynchium venkataramani
100/100
Anterhynchium mellyi
100/100
Anterhynchium abdominale
100/100
Rhynchium quinquecinctum
100/100
Rhynchium brunneum
100/100
Dirhynchium flavomarginatum
100/100
Dirhynchium coracinum
Dolichovespula media
100/100
Vespa crabro
0.02
usco90_GHOST

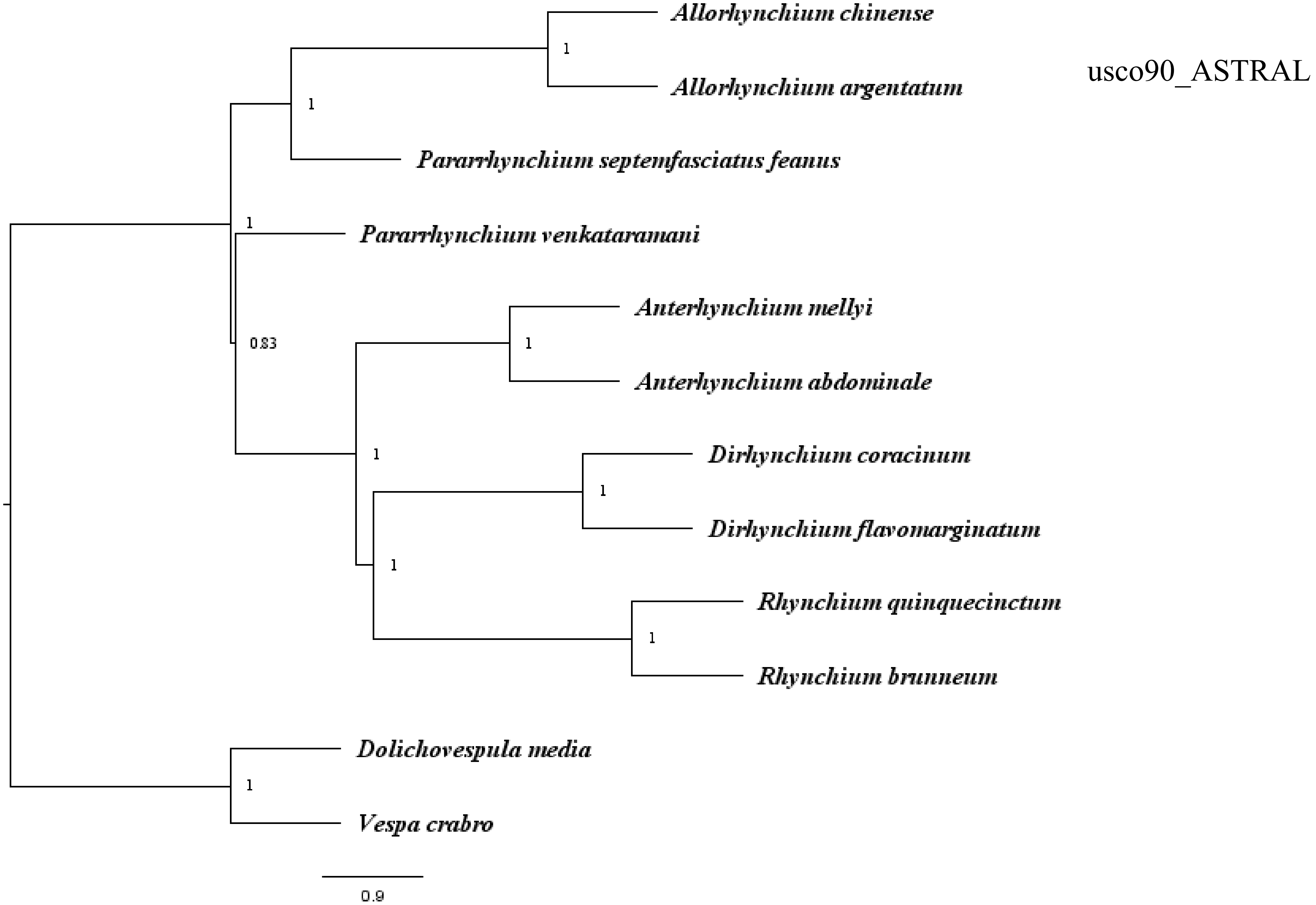

usco90_ASTRAL
Allorhynchium chinense
Allorhynchium argentatum
Pararrhynchium septemfasciatus feanus
Pararrhynchium venkataramani
Anterhynchium mellyi
Anterhynchium abdominale
Dirhynchium coracinum
Dirhynchium flavomarginatum
Rhynchium quinquecinctum
Rhynchium brunneum
Dolichovespula media
Vespa crabro
1
1
1
0.83
1
1
1
1
1
1
0.9

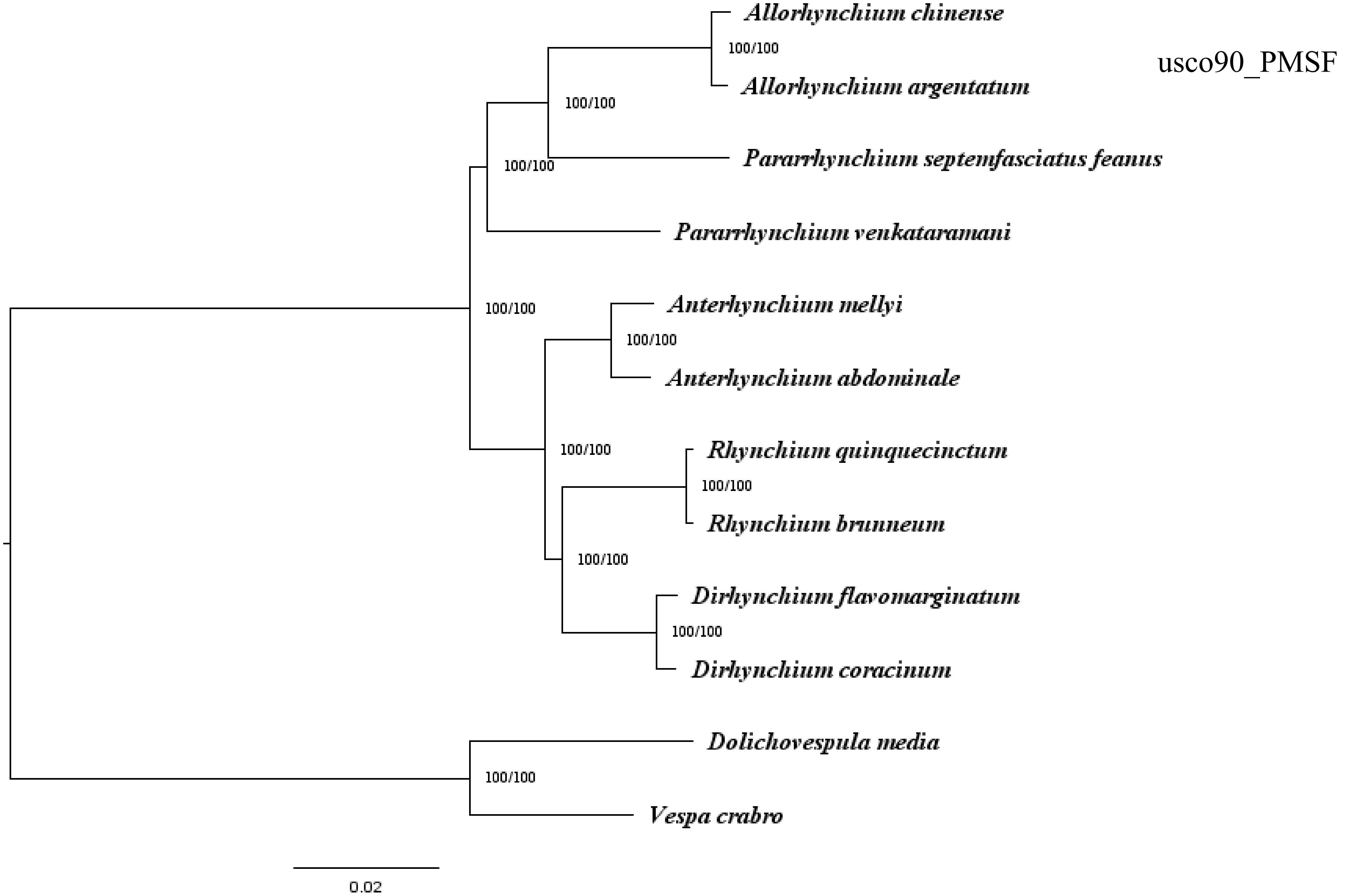
usco90_PMSF
Allorhynchium chinense
100/100
Allorhynchium argentatum
100/100
Pararrhynchium septemfasciatus feanus
100/100
Pararrhynchium venkataramani
100/100
Anterhynchium mellyi
100/100
Anterhynchium abdominale
100/100
Rhynchium quinquecinctum
100/100
Rhynchium brunneum
100/100
Dirhynchium flavomarginatum
100/100
Dirhynchium coracinum
Dolichovespula media
100/100
Vespa crabro
0.02

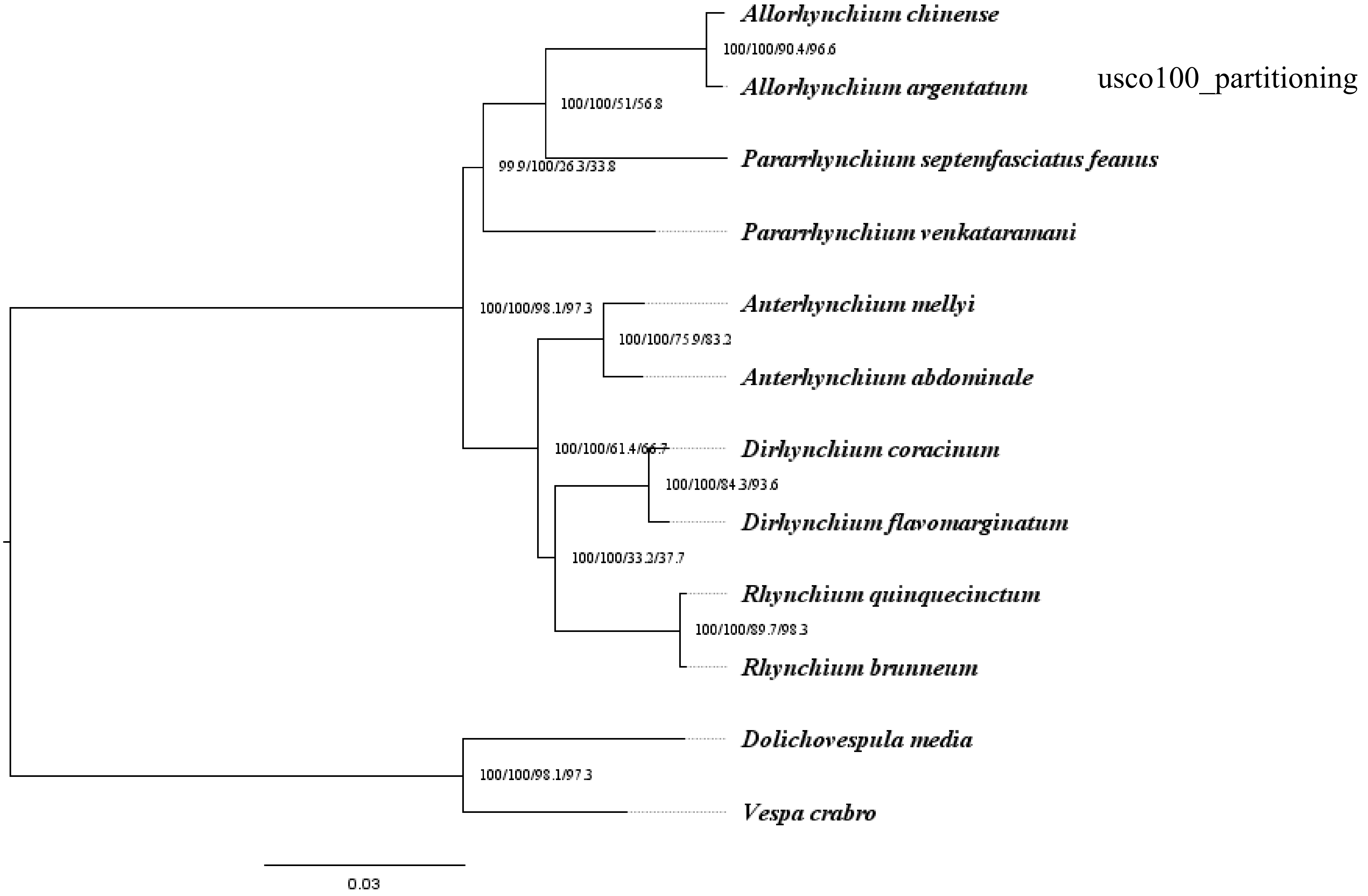
Allorhynchium chinense
100/100/90.4/96.6
Allorhynchium argentatum
usco100_partitioning
100/100/51/56.8
Pararrhynchium septemfasciatus feanus
99.9/100/26.3/33.8
Pararrhynchium venkataramani
100/100/98.1/97.3
Anterhynchium mellyi
100/100/75.9/83.2
Anterhynchium abdominale
100/100/61.4/66.7
Dirhynchium coracinum
100/100/84.3/93.6
Dirhynchium flavomarginatum
100/100/33.2/37.7
Rhynchium quinquecinctum
100/100/89.7/98.3
Rhynchium brunneum
Dolichovespula media
100/100/98.1/97.3
Vespa crabro
0.03

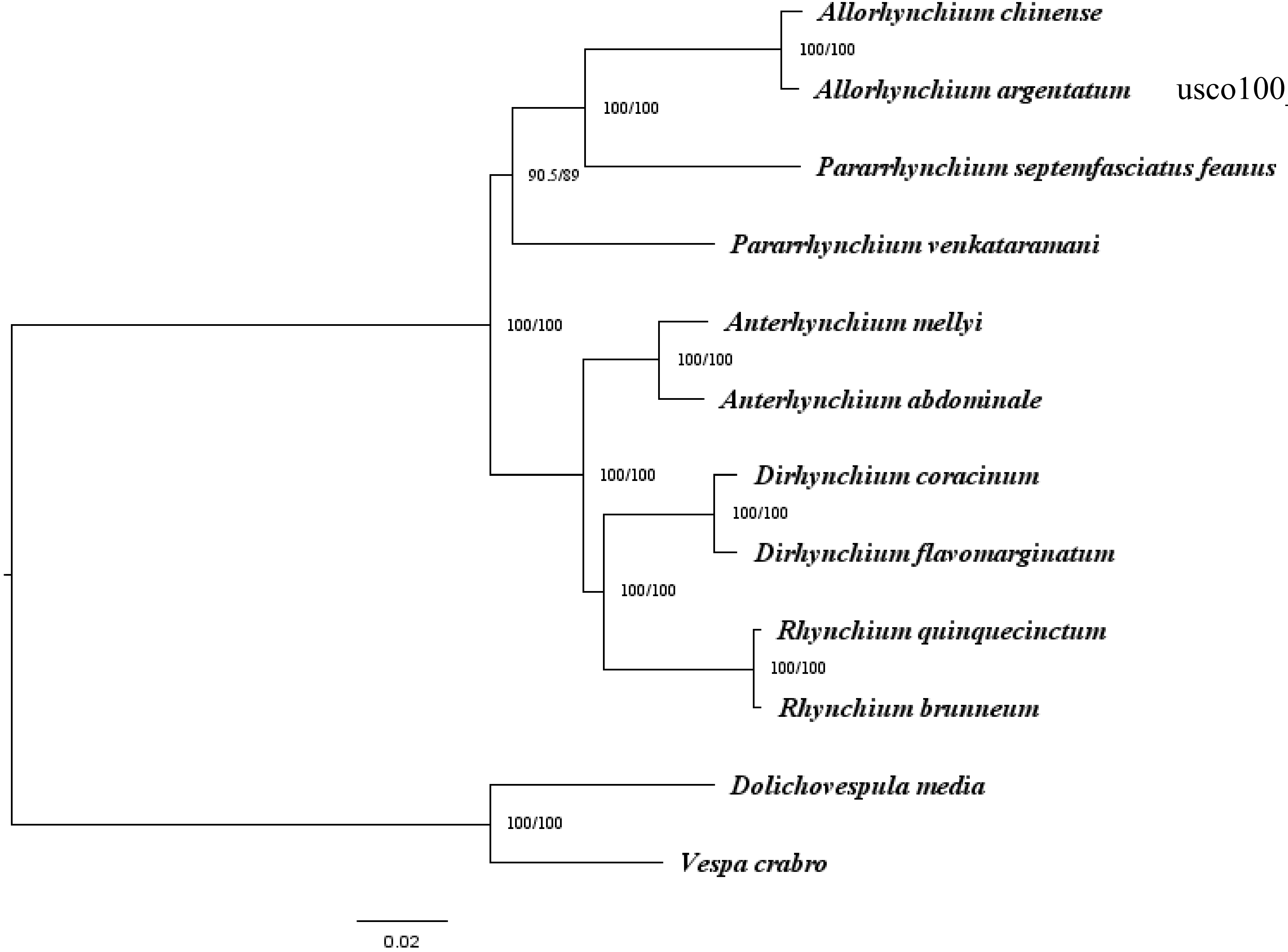

usco100_GHOST

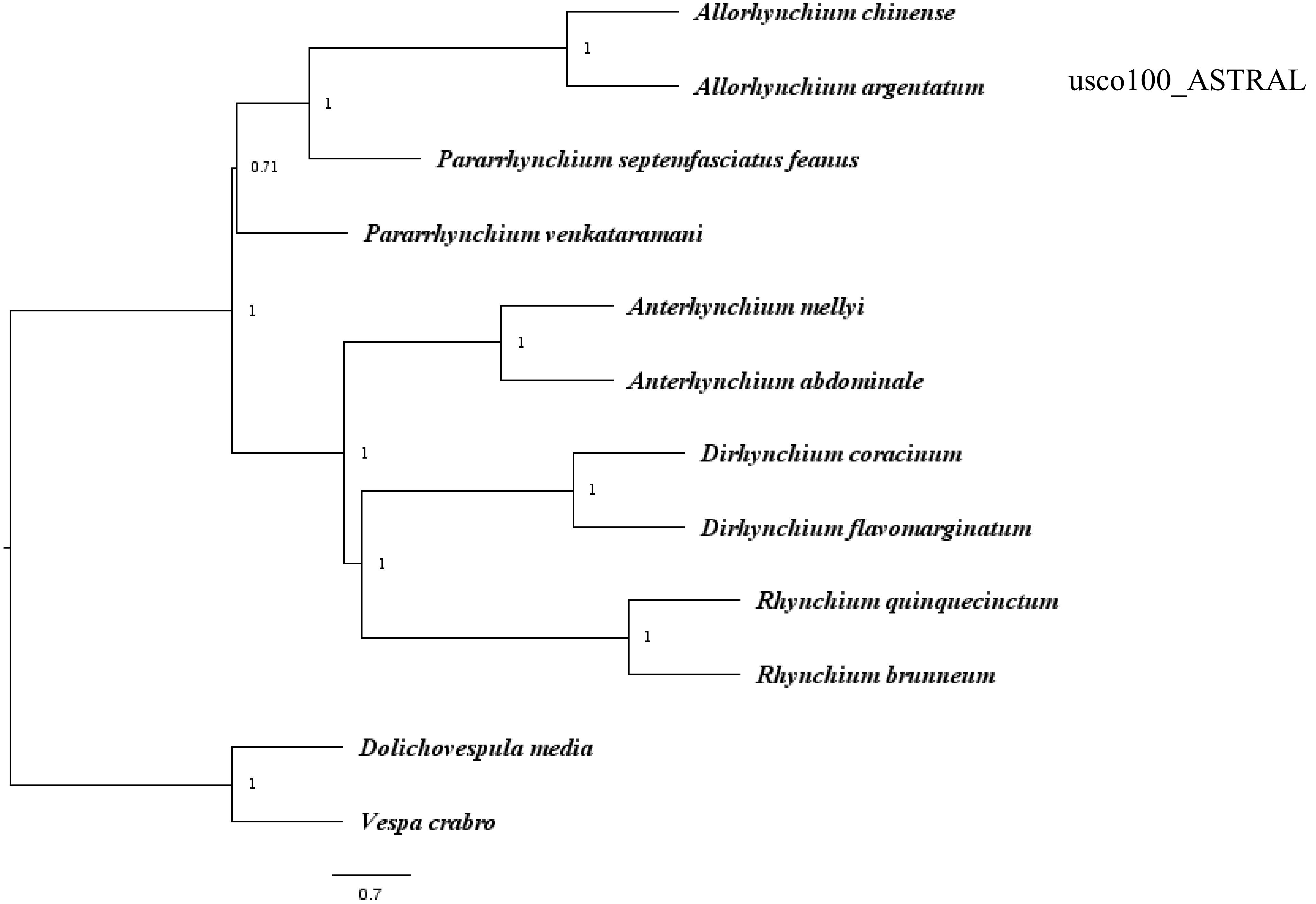

Allorhynchium chinense
Allorhynchium argentatum
Pararrhynchium septemfasciatus feanus
Pararrhynchium venkataramani
Anterhynchium mellyi
Anterhynchium abdominale
Dirhynchium coracinum
Dirhynchium flavomarginatum
Rhynchium quinquecinctum
Rhynchium brunneum
Dolichovespula media
Vespa crabro
1
1
0.71
1
1
1
1
1
1
1
0.7
usco100_ASTRAL

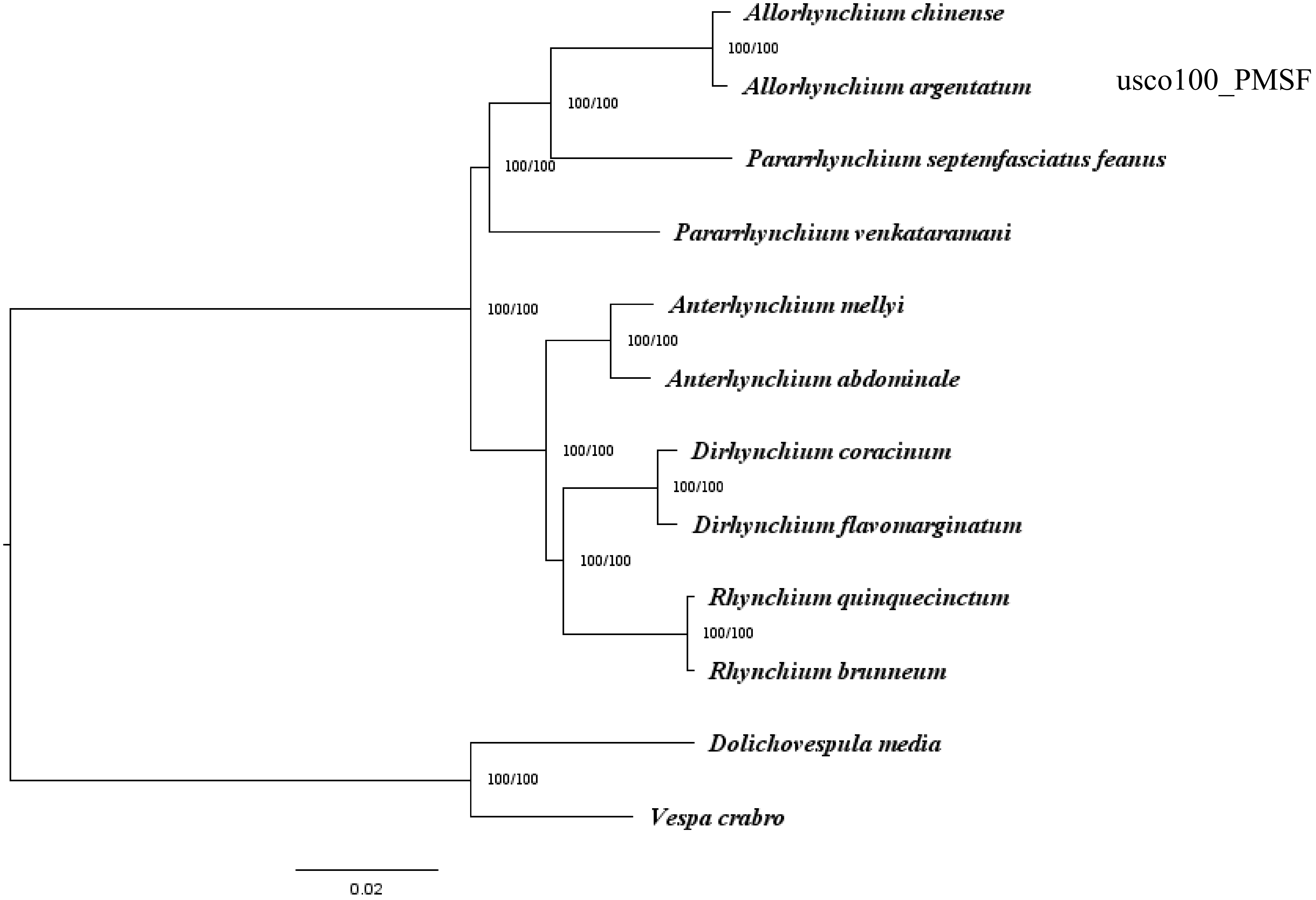

usco100_PMSF
Allorhynchium chinense
Allorhynchium argentatum
Pararrhynchium septemfasciatus feanus
Pararrhynchium venkataramani
Anterhynchium mellyi
Anterhynchium abdominale
Dirhynchium coracinum
Dirhynchium flavomarginatum
Rhynchium quinquecinctum
Rhynchium brunneum
Dolichovespula media
Vespa crabro
100/100
0.02

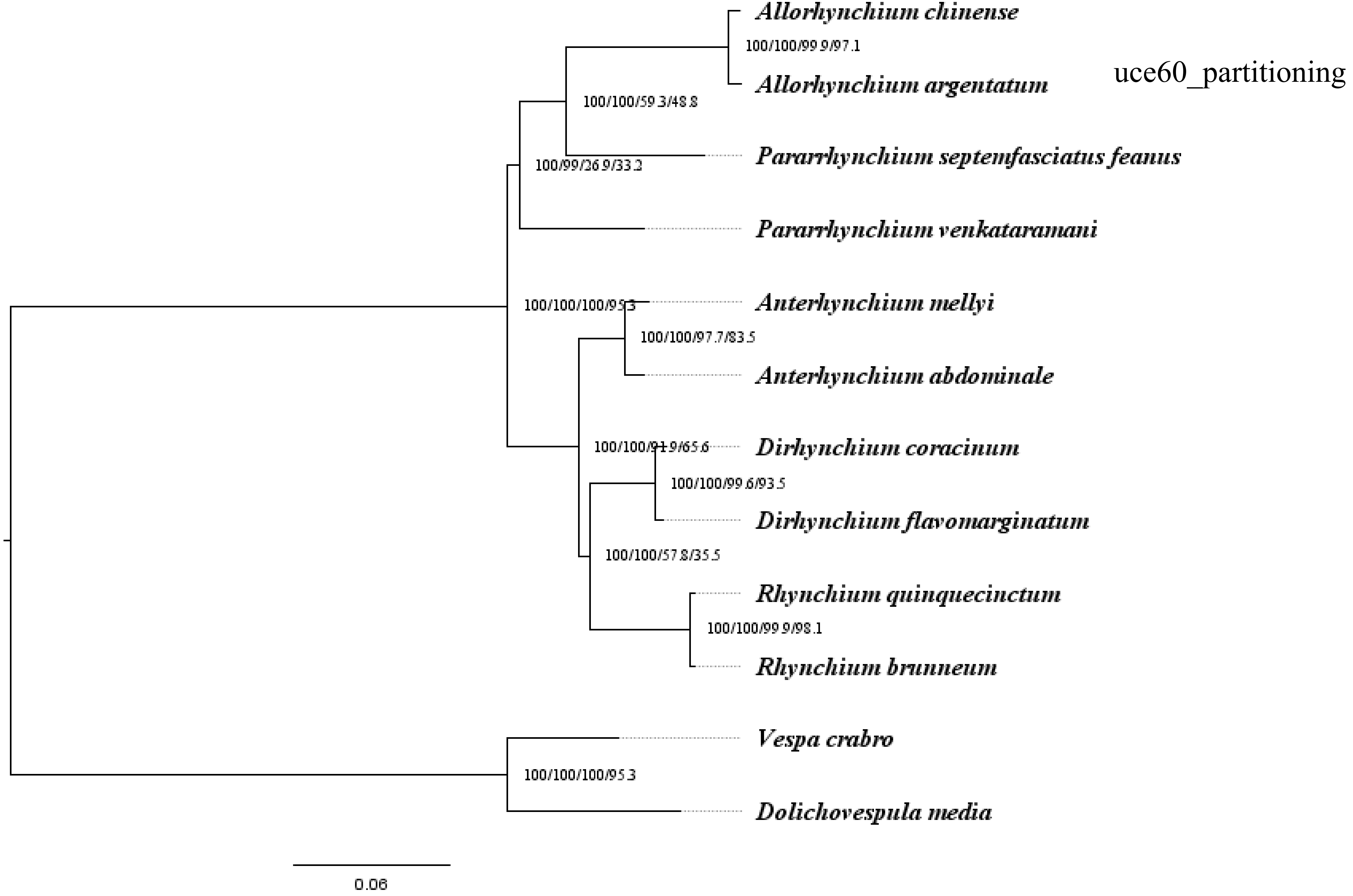

uce60_partitioning
Allorhynchium chinense
100/100/99.9/97.1
Allorhynchium argentatum
100/100/59.3/48.8
Pararrhynchium septemfasciatus feanus
100/99/26.9/33.2
Pararrhynchium venkataramani
100/100/100/95.3
Anterhynchium mellyi
100/100/97.7/83.5
Anterhynchium abdominale
100/100/91.9/65.6
Dirhynchium coracinum
100/100/99.6/93.5
Dirhynchium flavomarginatum
100/100/57.8/35.5
Rhynchium quinquecinctum
100/100/99.9/98.1
Rhynchium brunneum
Vespa crabro
100/100/100/95.3
Dolichovespula media
0.06

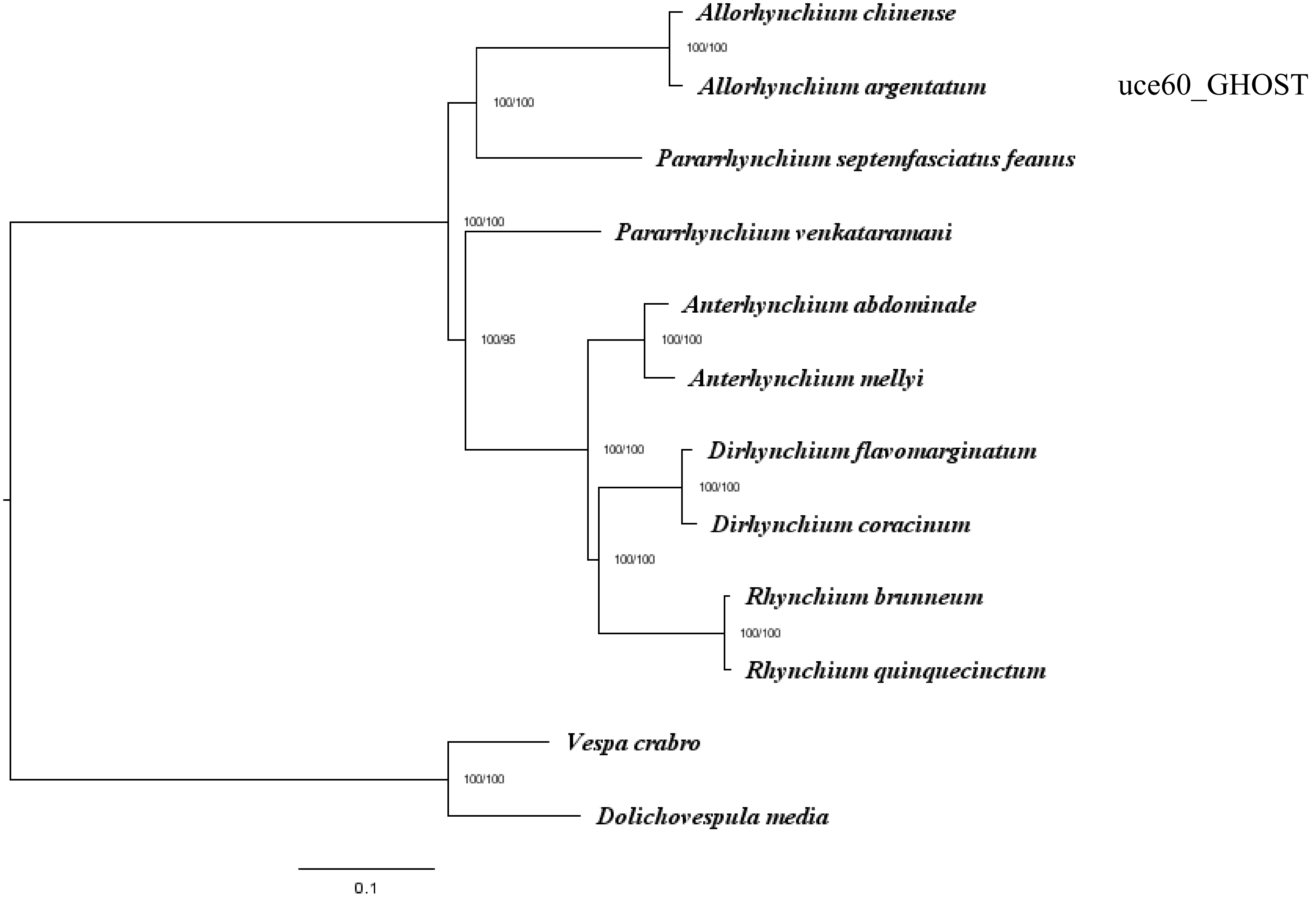
Allorhynchium chinense
100/100
Allorhynchium argentatum
uce60_GHOST
100/100
Pararrhynchium septemfasciatus feanus
100/100
Pararrhynchium venkataramani
Anterhynchium abdominale
100/95
100/100
Anterhynchium mellyi
100/100
Dirhynchium flavomarginatum
100/100
Dirhynchium coracinum
100/100
Rhynchium brunneum
100/100
Rhynchium quinquecinctum
Vespa crabro
100/100
Dolichovespula media
0.1

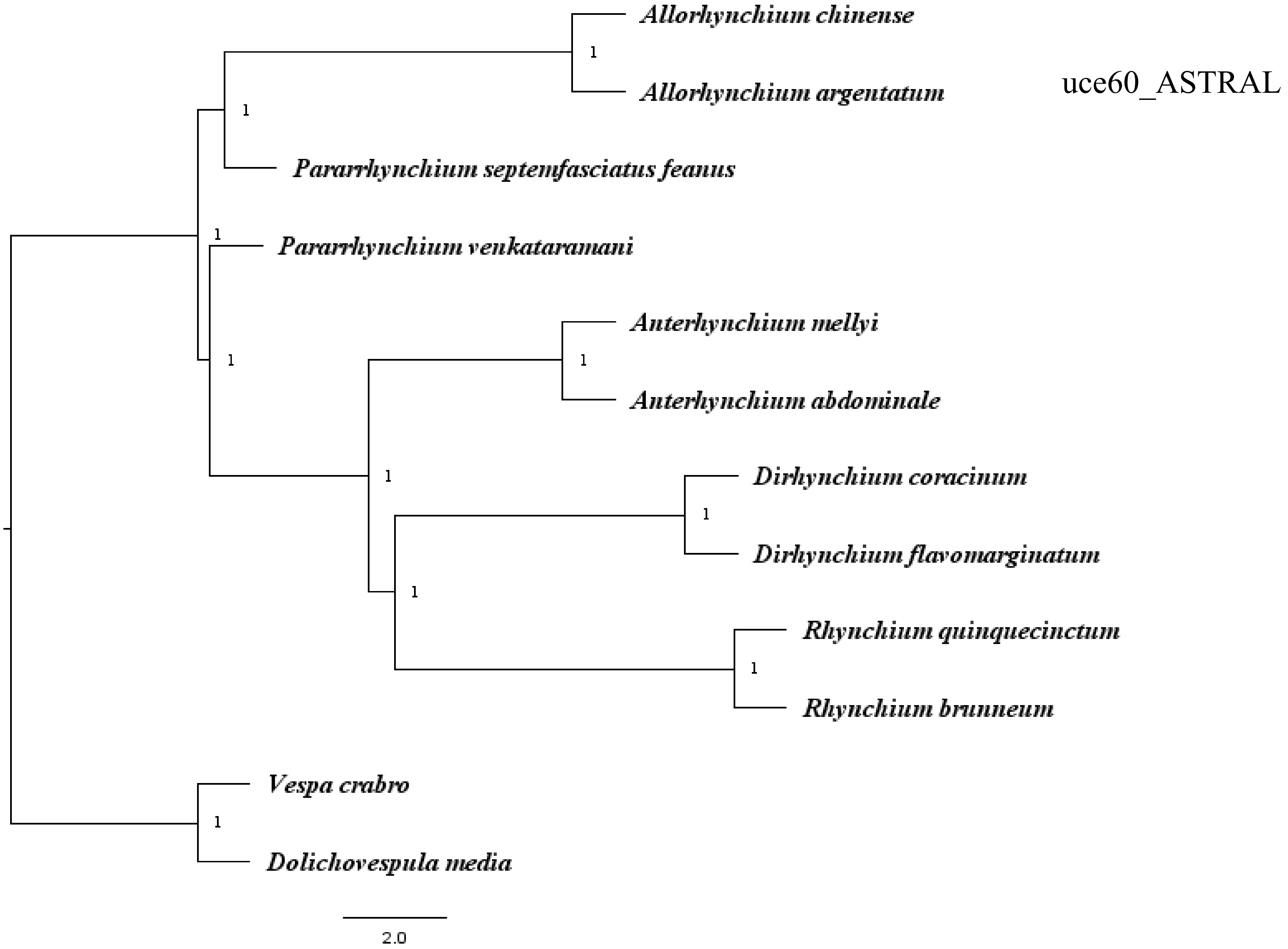
uce60_ASTRAL
Allorhynchium chinense
Allorhynchium argentatum
Pararrhynchium septemfasciatus feanus
Pararrhynchium venkataramani
Anterhynchium mellyi
Anterhynchium abdominale
Dirhynchium coracinum
Dirhynchium flavomarginatum
Rhynchium quinquecinctum
Rhynchium brunneum
Vespa crabro
Dolichovespula media
1
2.0

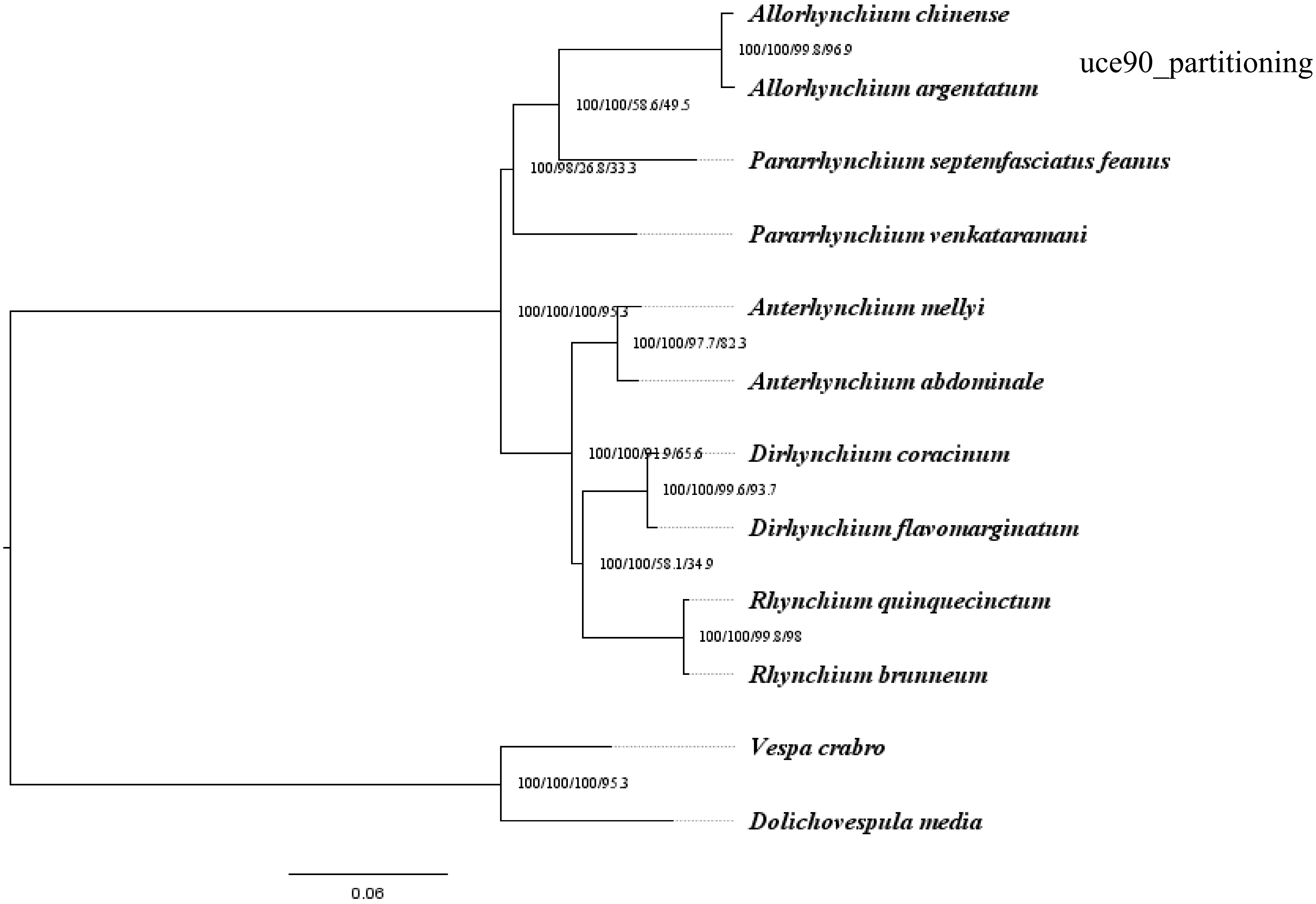
Allorhynchium chinense
100/100/99.8/96.9
uce90_partitioning
Allorhynchium argentatum
100/100/58.6/49.5
Pararrhynchium septemfasciatus feanus
100/98/26.8/33.3
Pararrhynchium venkataramani
Anterhynchium mellyi
100/100/100/95.3
100/100/97.7/82.3
Anterhynchium abdominale
Dirhynchium coracinum
100/100/91.9/65.6
100/100/99.6/93.7
Dirhynchium flavomarginatum
100/100/58.1/34.9
Rhynchium quinquecinctum
100/100/99.8/98
Rhynchium brunneum
Vespa crabro
100/100/100/95.3
Dolichovespula media
0.06

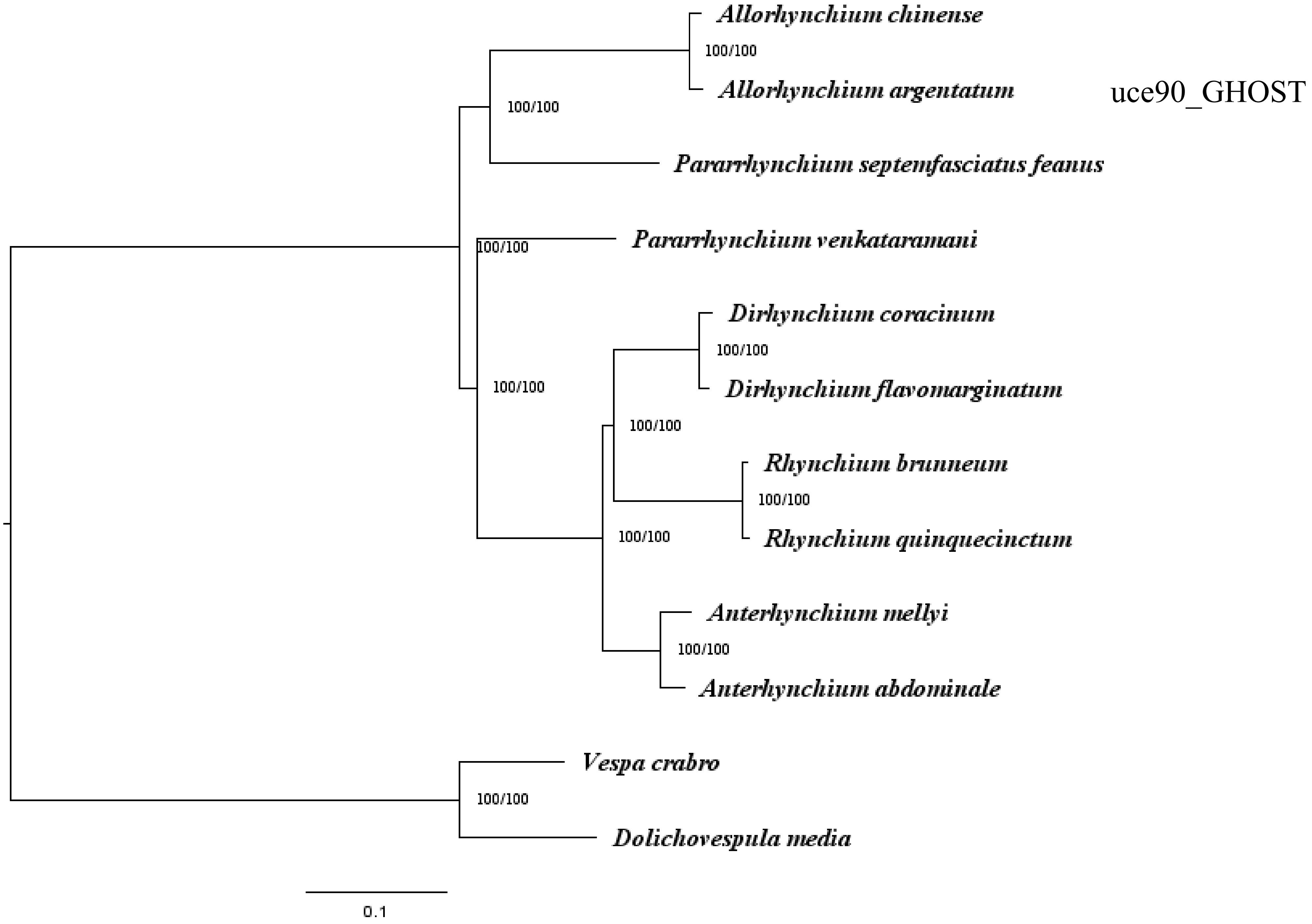
Allorhynchium chinense
100/100
Allorhynchium argentatum
uce90_GHOST
100/100
Pararrhynchium septemfasciatus feanus
Pararrhynchium venkataramani
100/100
Dirhynchium coracinum
100/100
Dirhynchium flavomarginatum
100/100
100/100
Rhynchium brunneum
100/100
Rhynchium quinquecinctum
100/100
Anterhynchium mellyi
100/100
Anterhynchium abdominale
Vespa crabro
100/100
Dolichovespula media
0.1

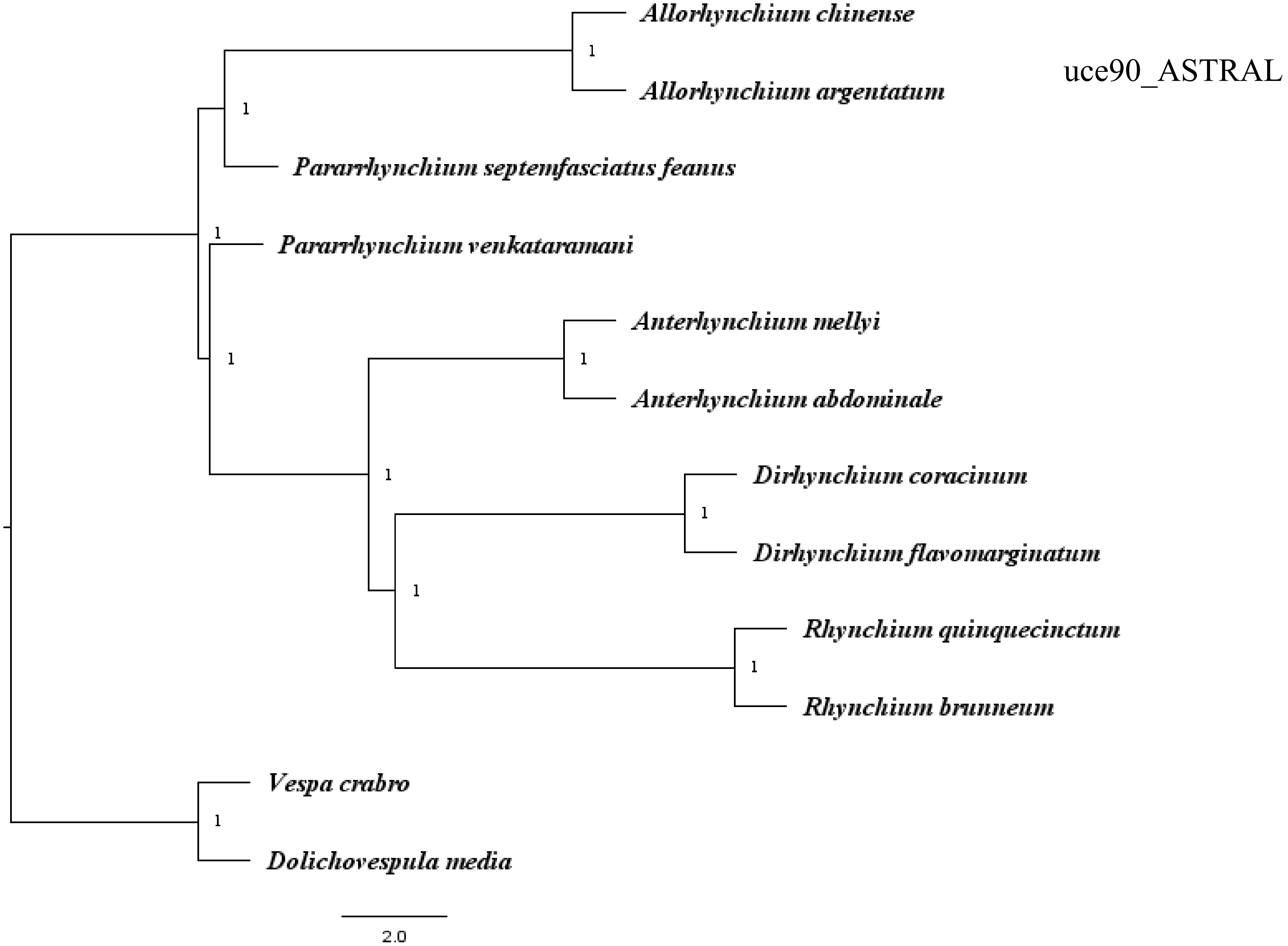
Allorhynchium chinense
Allorhynchium argentatum
Pararrhynchium septemfasciatus feanus
Pararrhynchium venkataramani
Anterhynchium mellyi
Anterhynchium abdominale
Dirhynchium coracinum
Dirhynchium flavomarginatum
Rhynchium quinquecinctum
Rhynchium brunneum
Vespa crabro
Dolichovespula media
uce90_ASTRAL
2.0

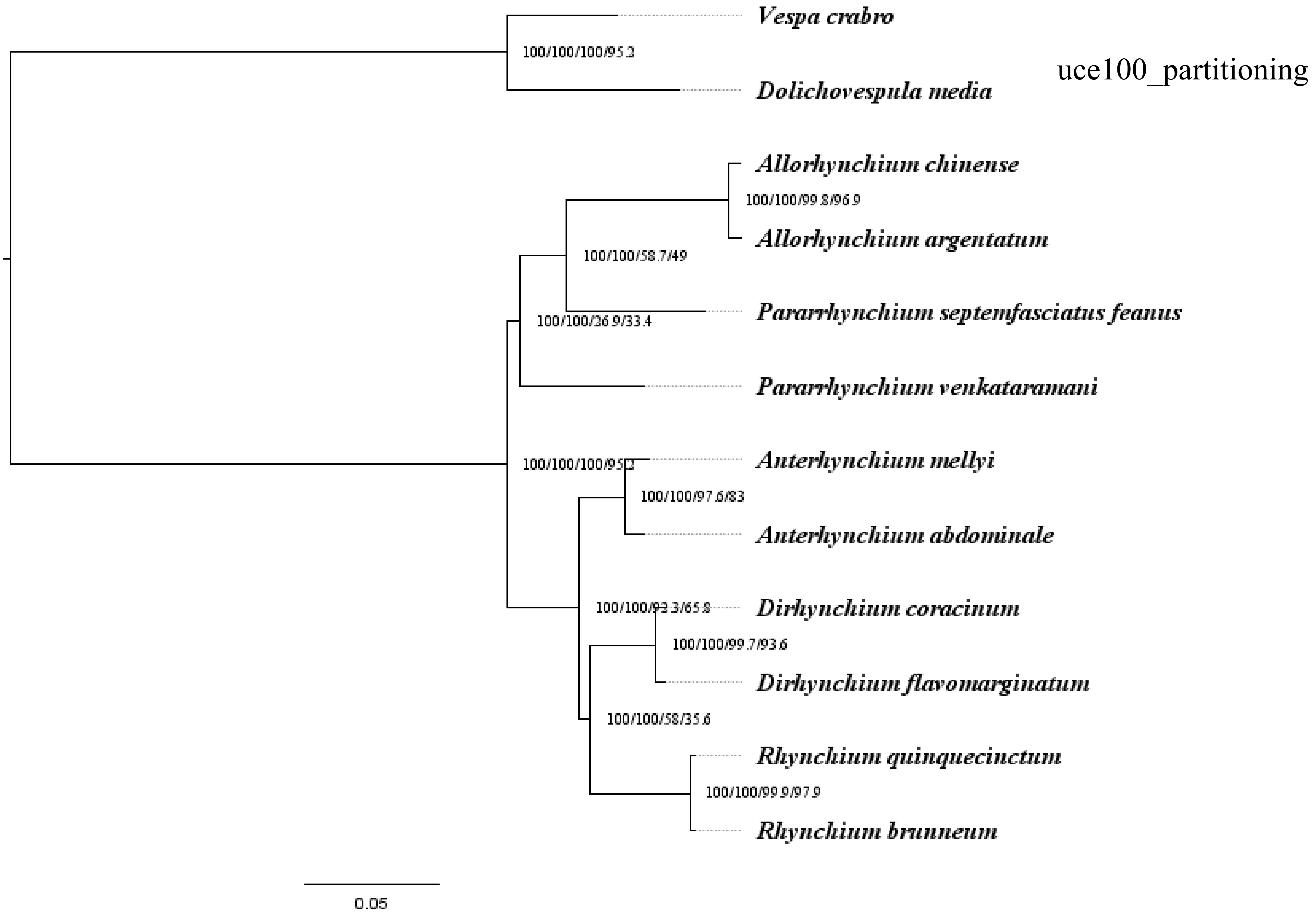
uce100_partitioning
Vespa crabro
Dolichovespula media
100/100/100/95.2
Allorhynchium chinense
Allorhynchium argentatum
100/100/99.8/96.9
100/100/58.7/49
Pararrhynchium septemfasciatus feanus
100/100/26.9/33.4
Pararrhynchium venkataramani
100/100/100/95.2
Anterhynchium mellyi
100/100/97.6/83
Anterhynchium abdominale
100/100/92.3/65.8
Dirhynchium coracinum
100/100/99.7/93.6
Dirhynchium flavomarginatum
100/100/58/35.6
Rhynchium quinquecinctum
100/100/99.9/97.9
Rhynchium brunneum
0.05

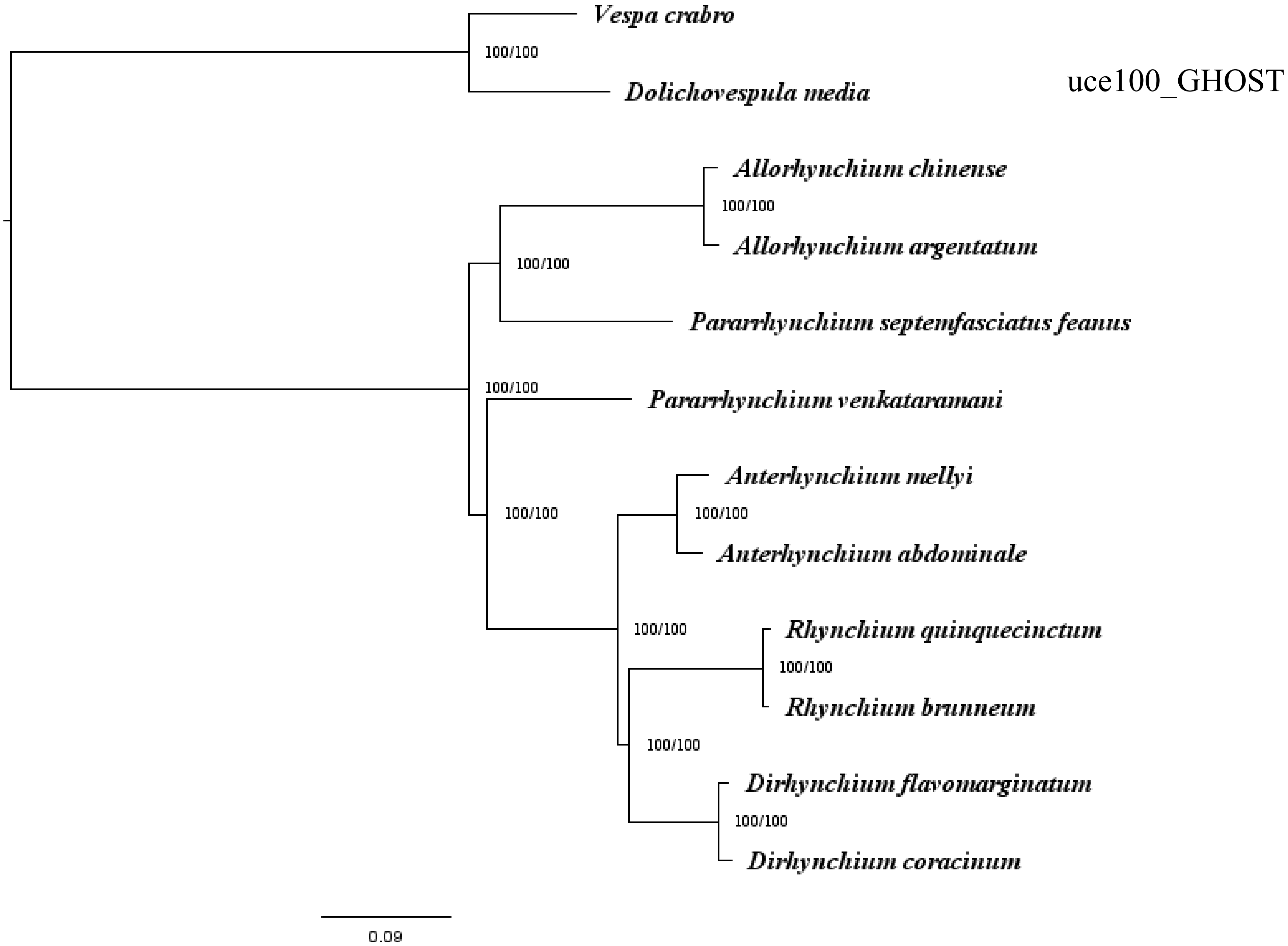
Vespa crabro
100/100
Dolichovespula media
uce100_GHOST
Allorhynchium chinense
100/100
Allorhynchium argentatum
100/100
Pararrhynchium septemfasciatus feanus
100/100
Pararrhynchium venkataramani
Anterhynchium mellyi
100/100
100/100
Anterhynchium abdominale
100/100
Rhynchium quinquecinctum
100/100
Rhynchium brunneum
100/100
Dirhynchium flavomarginatum
100/100
Dirhynchium coracinum
0.09

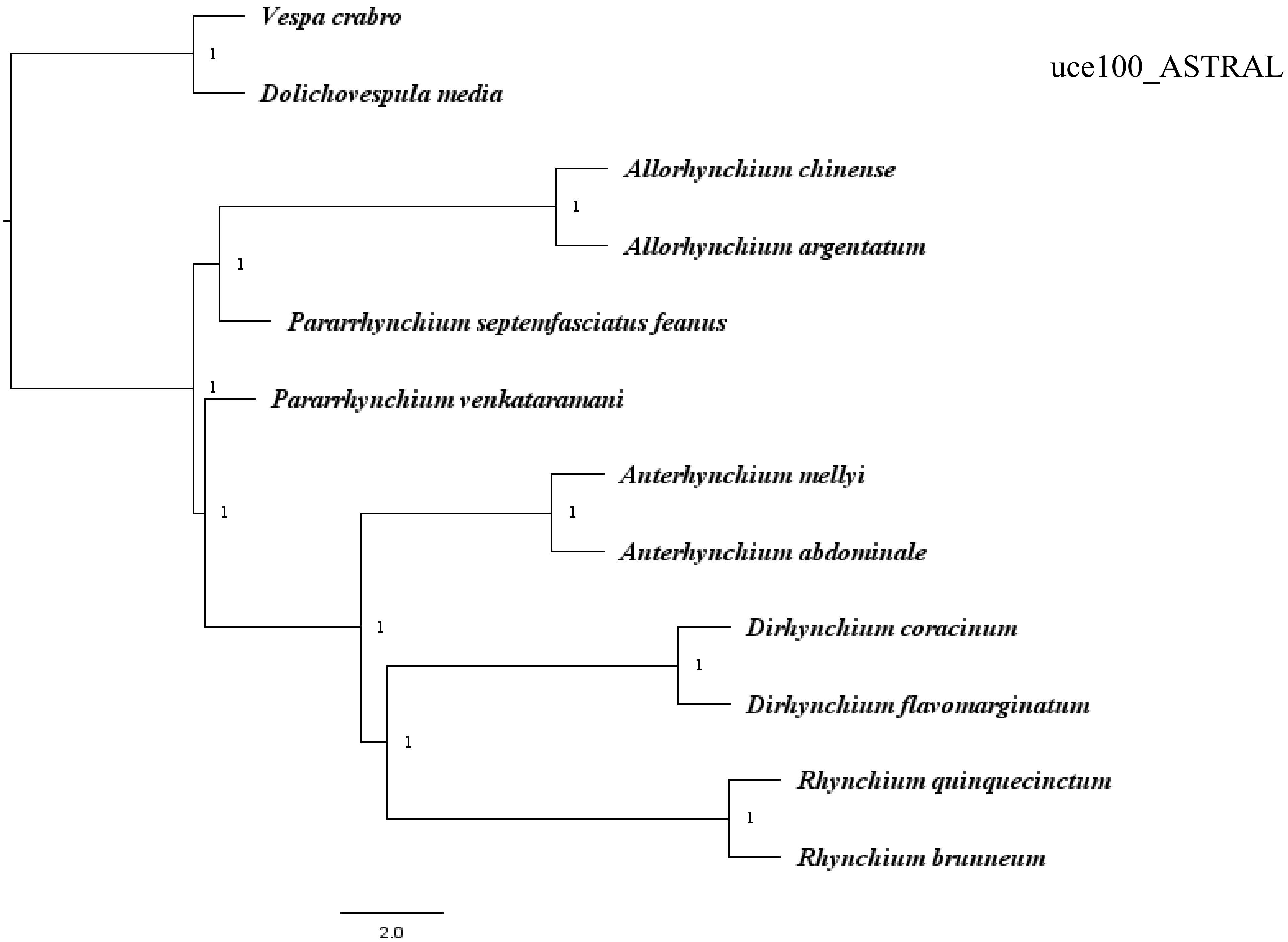
Vespa crabro
Dolichovespula media
Allorhynchium chinense
Allorhynchium argentatum
Pararrhynchium septemfasciatus feanus
Pararrhynchium venkataramani
Anterhynchium mellyi
Anterhynchium abdominale
Dirhynchium coracinum
Dirhynchium flavomarginatum
Rhynchium quinquecinctum
Rhynchium brunneum
uce100_ASTRAL
1
1
1
1
1
1
1
1
1
1
2.0
